# Impact of glyphosate and glyphosate-based herbicides on phyllospheric *Methylobacterium*

**DOI:** 10.1186/s12870-024-04818-x

**Published:** 2024-02-19

**Authors:** Daniel Palberg, Emma Kaszecki, Chetan Dhanjal, Anna Kisiała, Erin N. Morrison, Naomi Stock, R. J. Neil Emery

**Affiliations:** 1 Environmental and Life Sciences Graduate Program, Trent University, 1600 West Bank Drive, Peterborough, ON K9L 0G2 Canada; 2 Department of Biological Sciences, Carnegie Mellon University, 5000 Forbes Ave, Pittsburgh, PA 15213 USA; 3 Department of Biology, Trent University, 1600 West Bank Drive, Peterborough, ON K9L 0G2 Canada; 4 Water Quality Centre, Trent University, 1600 West Bank Drive, Peterborough, ON K9L 0G2 Canada

**Keywords:** Agrichemicals, Glyphosate, Surfactant, Plant growth promoting bacteria, Phyllosphere, WeatherMax®, Transorb®

## Abstract

Symbiotic *Methylobacterium* comprise a significant portion of the phyllospheric microbiome, and are known to benefit host plant growth, development, and confer tolerance to stress factors. The near ubiquitous use of the broad-spectrum herbicide, glyphosate, in farming operations globally has necessitated a more expansive evaluation of the impacts of the agent itself and formulations containing glyphosate on important components of the plant phyllosphere, including *Methylobacterium*.

This study provides an investigation of the sensitivity of 18 strains of *Methylobacterium* to glyphosate and two commercially available glyphosate-based herbicides (GBH). Nearly all strains of *Methylobacterium* showed signs of sensitivity to the popular GBH formulations *WeatherMax*® and *Transorb*® in a modified Kirby Bauer experiment. However, exposure to pure forms of glyphosate did not show a significant effect on growth for any strain in both the Kirby Bauer test and in liquid broth, until polysorbate-20 (Tween20) was added as a surfactant. Artificially increasing membrane permeability through the introduction of polysorbate-20 caused a 78–84% reduction in bacterial cell biomass relative to controls containing glyphosate or high levels of surfactant only (0–9% and 6–37% reduction respectively). Concentrations of glyphosate as low as 0.05% w/v (500 µg/L) from both commercial formulations tested, inhibited the culturability of *Methylobacterium* on fresh nutrient-rich medium.

To better understand the compatibility of important phyllospheric bacteria with commercial glyphosate-based herbicides, this study endeavours to characterize sensitivity in multiple strains of *Methylobacterium*, and explore possible mechanisms by which toxicity may be induced.

## Introduction

**Fig. 1:**
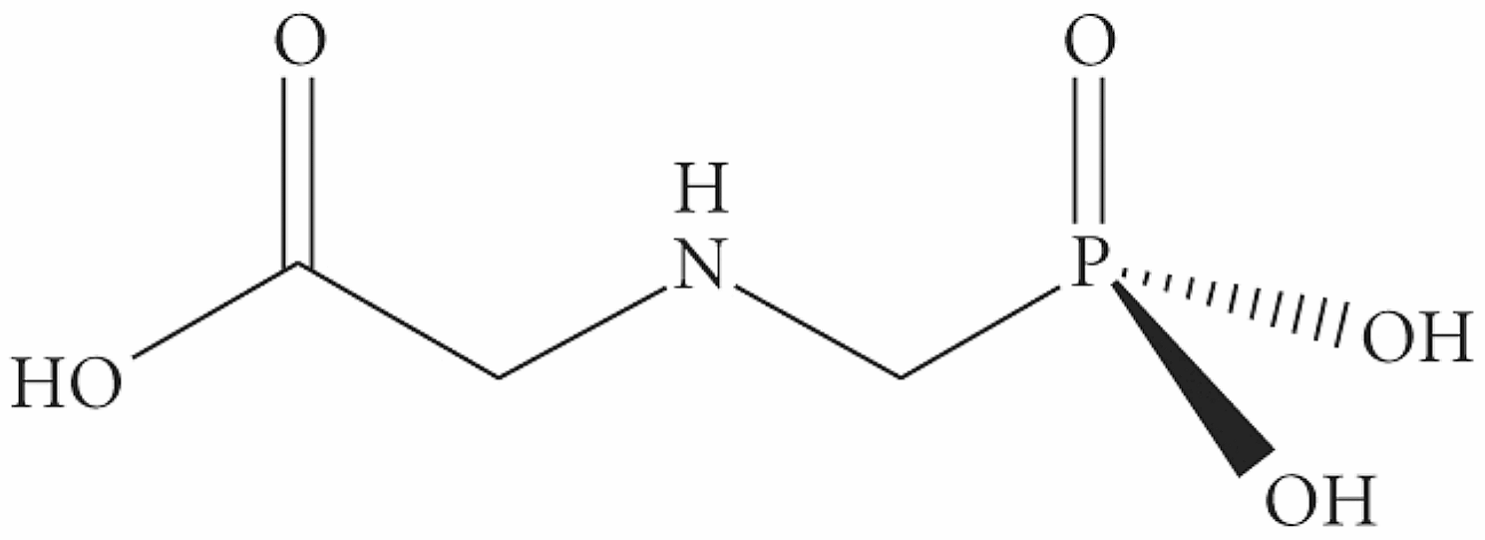
chemical structure of glyphosate [N-(phosphonomethyl)glycine]

In contemporary farming, the removal of problematic weeds is critical to minimizing crop loss caused by resource competition, and reduce contamination during harvest. In contrast to manual and mechanical weeding, the use of herbicides offers a highly cost effective and resource-efficient method of obtaining control over opportunistic vegetation. Some herbicidal agents introduced since the mid 1900s widely recognized for their performance include 2,4-dichlorophenoxyacetic acid (2,4-D), atrazine, pendimethalin, and dicamba [1]. While highly effective, these compounds have been associated with a wide array of unfavourable characteristics. For example, atrazine contaminates ground water sources due to low soil binding affinity, and dicamba is considered carcinogenic to mammals and toxic to aquatic life [1]. With an array of non-target effects observed in first-generation herbicides, the market desire for an herbicidal agent with an improved toxicological profile was substantial. It would not be until 1970 that the herbicidal activity of glyphosate was characterized by John E. Franz, despite Henri Martin discovering the molecule in 1950. Chemically, glyphosate [N-(phosphonomethyl)glycine] is an organophosphate with the chemical formula C_3_H_8_NO_5_P, a molecular weight of 169.073 g·mol^− 1^. The compound is synthesized through the oxidative coupling of methylphosphonic acid and a glycine residue (Fig. 1).

By 1971, the Monsanto corporation patented glyphosate (U.S.A. Pat.No. 3,799,758) and marketed the compound in various formulations under the trade name RoundUp®. Shortly after RoundUp® products were released to market, glyphosate quickly became the leading herbicide applied by volume in the world, increasing from 1.4 million pounds in 1974 to 40 million pounds in 1995 [2]. Much of the success of glyphosate is owed to its broad-spectrum herbicidal activity, rapid absorption through leaf tissues, and relatively high soil binding affinity [3–6]. Collectively, these attributes lower the required frequency of application and reduce the number of different products necessary to achieve adequate vegetation control. High binding affinity for soil also limits leachability of the active ingredient into groundwater and nearby aquatic ecosystems. In commercial formulations, additives for storage stability and adjuvants including surfactants, anti-foaming compounds, and buffering agents work in concert to enhance the activity of glyphosate by improving dispersal and persistence on, and permeability through, plant tissues. Once absorbed into a plant, glyphosate rapidly translocates to the roots, developing reproductive organs, and meristematic tissues which further heightens its potency [7].

The mechanism of action (MoA) of glyphosate is based in its ability to disrupt 5-enolpyruvylshikimate-3-phosphate synthase (EPSPS). EPSPS is a monomeric enzyme belonging to the transferase family and is responsible for executing a critical step in the shikimate pathway. The shikimate pathway is the primary means for aromatic amino acid (AAA) production (tryptophan [Tyr]; tyrosine [Tyr]; and phenylalanine [Phe]) in plants, fungi, and bacteria, and acts as a biosynthetic shunt linking primary and secondary metabolism. There are currently two known isozymes of EPSPS; EPSPS class I, and EPSPS class II. Both forms catalyze the addition of phosphoenolpyruvate (PEP) to shikimate-3-phosphate and release 5-enolpyruvylshikimate-3-phosphate (EPSP) as the terminal product. Where EPSPS I (EPSPS: EC 2.5.1.19) is highly sensitive to glyphosate which inhibits binding of PEP, EPSPS II is not susceptible. Interestingly, while most plants, fungi, and gram-negative bacteria share EPSPS I, the resistant CP4 EPSP synthase isozyme was first isolated from a unique strain of *Agrobacterium* found in a wastewater line at a glyphosate manufacturing site [8]. Through the inhibition of the glyphosate-sensitive EPSPS I and subsequent inactivation of the shikimate pathway, AAA biosynthesis is impaired. In plants, the AAA are essential for the formation of structural components of the cell and participate in enzyme activation as they often facilitate protein folding. The AAA also serve as precursors to secondary metabolites (flavonoids, stilbenes, phenylpropanoids, alkaloids), and several important phytohormones (salicylate, auxin) [9, 10]. AAA biosynthesis in bacteria fulfill similar structural and non-protein roles, including the development of antibiotic and antimycotic compounds [11–13].

Lacking shikimate machinery should leave humans and wildlife generally unaffected by glyphosate. Despite a plethora of independent studies examining the toxic potential of glyphosate in a variety of models including rats [14–17], zebra fish [18–21], and fruit fly [22–24], investigation of the effects of glyphosate and GBH’s on microorganisms is comparatively low, and this has remained especially so with respect to plant-associated bacteria. To date, investigations regarding the effects of glyphosate on the phyllosphere are mixed, and focus greatly on rhizosphere bacteria over those in the shoot system [25, 29]. This has likely been driven by research concerning the chemical behaviour of glyphosate in the soil in an effort to establish key physiochemical properties such as persistence, bioaccumulation, migration, and leaching potential. However, this has resulted in little actually being known about how glyphosate may affect important microbial members of the phyllosphere.

Referring to the collection of microorganisms including bacteria, viruses, protozoa, archaea, and algae that inhabit plants, the plant microbiome plays crucial roles in the health and development of their host-plant. Similar to the human microbiome, microorganisms may establish a relationship with the host that varies on the scale between mutualistic to parasitic. To date, investigations regarding the effects of glyphosate on the plant microbiome are mixed and focus largely on the rhizosphere, leaving little understanding of the effects of glyphosate on the aerial plant microbiome [25–29].

The *Methylobacterium* is a genus of bacteria which often comprise a large part of the natural microbiota that inhabit plants [30] and are so ubiquitous in nature that they have also been isolated from a wide array of sources including soil [31], air [31], water [31], humans [32], food [33], and spacecraft [34]. In addition to theories suggesting that microbial colonization is motivated by methanol emissions produced from cell wall remodelling [35–38], *Methylobacterium* spp. also actively play important roles in plant growth promotion. Several strains have been characterized as plant growth-promoting bacteria (PGPB) based on their ability to synthesize high quantities of growth-enhancing phytohormones including cytokinins (CKs) [39–42], while others uniquely facilitate nitrogen-fixation as is the case with *M. nodulans* [43, 44]. Significant tolerance to chlorine exposure [31], unfavourable salinity, pH, and temperature [40] are also documented traits of several *Methylobacterium* species, along with their ability to utilize both common carbon sources like carbohydrates in addition to oxidizing several single-carbon molecules including methanol, methylamine, and formaldehyde [32, 45, 46]. *Methylobacterium* are aerobic, gram-negative, facultative methylotrophs that use single-carbon compounds to grow, although several species have adaptations that allow the use of C2 and C3 compounds as well [38]. A distinct pink pigmentation is a frequently recognizable characteristic of *Methylobacterium*, however some exceptions to this have been established (*Methylobacterium jeotgali*) [33]. The presence of carotenoids have been suggested to cause the pink pigmentation which may in fact confer the ultraviolet (UV) and gamma radiation tolerance observed in earlier studies [30, 47–52]. Morphologically, *Methylobacterium* are rod-shaped, and exhibit polar growth.

While *Methylobacterium* spp. have been studied previously for suitability in a wide range of biotechnologies including bioremediation of environmental toxins [53, 54] and explosives [55], the activity of *Methylobacterium* within the plant microbiome, and subsequent influence on plant health has catalyzed interest for its agronomic potential [56–58]. Apart from improving the growth and yield of several crop types, select strains of Methylobacterium have also been determined to increase host-resilience against abiotic stressors including high temperatures and severe drought [33, 41, 46, 59–64]. Based on the presence of the requisite cellular machinery, *Methylobacterium* spp. may also be capable of host-protection through use of enzyme classes like glycosidases, pectinases, and chitinase to mount direct counterattacks against pathogenic fungi [56, 65–68]. Studies examining resistance to UV [48, 52] and gamma radiation [47], have prompted theories which suggest that the distinct pink-pigmentation of the *Methylobacterium* may not only contribute to the colour of some plant organs but afford enhanced protection from ionization radiation as well [50].

However, in the development of sustainable biofertilizers and crop protection products, compatibility with existing application techniques, equipment, and contemporary agrichemicals remains an important consideration for performance and marketability. The popularity of herbicidal products containing glyphosate has risen steadily since the invention of glyphosate-resistant (GR) cultivars of popular cash crops. Today, GR crops available on the commercial market include soybean, corn, canola, cotton, grass seed, and alfalfa [69]. Despite the availability of over 200 licensed varieties, over 60% of all soybeans planted each year belong to a GR cultivar [69]. However, in the same way that glyphosate eradicates weeds by disabling AAA biosynthesis, it may also be capable of blocking this essential function in important members of the plant microbiome including *Methylobacterium* spp. Moreover, little is known about the effects that *Methylobacterium* may have on the persistence, absorbability, and activity of glyphosate on target plants, should members across the genus tolerate or catabolize glyphosate.

Interestingly, in a 2010 patent filed by Monsanto Technology LLC (U.S.A. Pat.No. 7,771,736), glyphosate is rebranded as a highly-capable agent for the prevention and therapy of infectious disease caused by microorganisms. As with studies involving animal models, the existing sphere of research concerning the sensitivity of bacteria to glyphosate has remained equivocal. To the knowledge of the authors, this study is the first comprehensive investigation focused on the sensitivity of *Methylobacterium* to commercial herbicide products containing glyphosate.

## Materials and methods

### Chemicals and materials

Two commercial formulations of glyphosate were used in this study, RoundUp *WeatherMax*® (Bayer Agrichemicals; 48.8% potassium salt of glyphosate composition [w/v], PCP Reg. No. 27,487, LOT #MYWF1108AJ) and RoundUp *Transorb*® (Bayer Agrichemicals; 48.8% potassium salt of glyphosate composition [w/v], PCP Reg. No. 28,198, LOT #MWBK0516AJ), in addition to HPLC-grade glyphosate as potassium salt (Fisher Scientific, CAS: 70901-12-1) sourced for use as a positive control. Due to the proprietary nature of the commercial formulations, exact ingredients including the surfactants used and their exact concentrations could not be determined. Internal standards (> 99% purity) of glyphosate, aminomethylphosphonic acid (AMPA), and sarcosine (N-methyl glycine) were sourced from Millipore Sigma. The commercial formulations were selected based on their applicability to a wide range of crops and popularity in Canada. Analytical-grade formic acid (88%), and HPLC-grade acetonitrile (ACN), and methanol (MeOH) were obtained from Fisher Scientific. 50mL 0.2 μm conical filters used for filter sterilization of solutions were obtained from Fisher Scientific and ultra-pure water (18.2 MΩ-cm) was obtained from a Milli-Q system. Formaldehyde was sourced from Fisher Scientific as a 37% (w/w) stock solution, ammonium acetate reagent grade was obtained from Bio Basic Inc. at 98% purity, and anhydrous acetyl acetone (2,4-pentanedione) was sourced from Acros Organics at 99% purity. Sterile, blank paper disks (Oxoid, Fisher Scientific) of 6 mm diameter (< 1 mm thickness) were obtained to conduct a modified Kirby Bauer sensitivity assay. Polysorbate-20 (Tween20) was sourced from MiliporeSigma.

### Selection of bacterial strains & culture conditions

Preparation of bacterial cultures were conducted aseptically in a biological safety cabinet (BSC). Freeze-dried cultures of *Methylobacterium spp*. were obtained from four microbe collections: the Belgian Coordinated Collections of Microorganisms (BCCM/LMG), the Deutsche Sammlung von Mikroorganismen und Zellkulturen (DSMZ) [“German Collection of Microorganisms and Cell Cultures”], the Japan Collection of Microorganisms (JCM), and the National Institute of Technology and Evaluation’s (NITE) Biological Resource Center (NBRC). Strains were originally collected from different biological (living plants) and natural non-biological sources (soil, water, air). Information on strain taxonomy, origin, and known characteristics is provided in Table 1. The freeze-dried strains were revived in nutrient rich R2A broth (VWR, Mississauga, Canada) and cryogenically maintained as 15% (v/v) glycerol stocks at -80 °C. All nutrient-rich tryptic soy agar (TSA) plates were standardized to 1.5% (w/v) agar content, and 20 mL fill in sterile single-use petri dishes (Greiner bio-one, 94 × 16 mm).

**Table 1:** Inventory of *Methylobacterium* strains examined for glyphosate sensitivity

Species	Strain	Source of Isolation
M. *organophilum*	NBRC 103119(T)	*Pelargonium zonale*; phyllosphere
	NBRC 103121	*Begonia sp*.; phyllosphere
M. *phylospharae*	LMG 24361(T)	*Oryza sativa*; phyllosphere
M. *radiotolerans*	LMG 6379(T)	Forest soil
M. *jeotgali*	LMG 23639(T)	Traditional fermented seafood (*jeotgal*)
M. *oryzae*	LMG 23582	*Oryzae sativa*; phyllosphere
M. *oxalidis*	NBRC 107715	*Oxalis corniculata*; phyllosphere
M. *extorquens*	JCM 2805	Air
	JCM 2806	Garden soil, slough
	NBRC 103129	*Eucalyptus sp*.; phyllosphere
M. *gnaphali*	NBRC 107716	*Gnaphalium spicatum*; phyllosphere
M. *thiocyanatum*	NBRC 103128	*Mesenbryanthemum sp*.; phyllosphere
M. *zatmanii*	LMG 6087	-
M. *thiocyanatum*	JCM 10893	*Allium aflatuense*; phyllosphere
M. *rhodinum*	LMG 2275	Alder tree (*Alnus*); phyllosphere
M. *iners*	JCM 16407	Air
*Methylobacterium spp*.	JCM 14674	*Oryza rufipogon*: phyllosphere
	DSM 23935	*Cardamine hirsuta;* phyllosphere
E. *coli*	NM 522	*-*

### Revival of cryogenic stocks

*Methylobacterium* strains were aseptically streaked on nutrient-rich tryptic soy agar (TSA) (Fisher Scientific) using a sterile loop. After 5 days of incubation at 27^o^C, single colonies were extracted from each plate and used to inoculate 50 mL of TSB liquid growth media in 150 mL vented flasks (Fisher Scientific; 0.22 μm) and maintained in a rotary incubator for 5 days (27^o^C and 110 RPM). When *Methylobacterium* cultures reached the late exponential/early stationary phase after approximately 6 days (OD_600_ = 0.6– 1.2, depending on strain, approximately 10^8^ CFU / mL) (*Genesys*™ 10s Visible Spectrophotometer, Thermo Fisher Scientific) nutrient minimum agar plates were inoculated from the TSB liquid media using a sterile loop and streak method. Selective minimum nutrient medium was prepared in accordance with the Deutsche Sammlung von Mikroorganismen und Zellkulturen (DSMZ) [“German Collection of Microorganisms and Cell Cultures”] recipe for *Methylobacterium* growth media (DSMZ Index 125): KNO_3_—1.00 g; MgSO_4_ × 7H_2_O—0.2 g; CaCl_2_ × 2H_2_O—0.02 g; Na_2_HPO_4_—0.23 g; NaH_2_PO_4_—0.07 g; FeSO_4_ × 7H_2_O—1.00 mg; CuSO_4_ × 5H_2_O – 5 µg; H_3_BO_3_—10 µg; MnSO_4_ × 5H_2_O – 10 µg; ZnSO_4_ × 7H_2_O—70 µg; MoO_3_ – 10 µg; H_2_O—1000 mL; CH_3_OH—5 mL; pH 6.8). Transfer to a minimum nutrient medium ensures high selectivity for *Methylobacterium* and reduced potential for contamination during the initial transfer from cryogenically preserved stock solutions. After 5 days of incubation at 27^o^C, single colonies of each bacterium were extracted from each plate and used to inoculate culture flasks, as necessary. Stock plates were sealed with parafilm and maintained at 4^o^C.

### Preparation of paper disks & optimization of loading volume

The Kirby Bauer sensitivity assay is a method for visually assessing sensitivity of microbial strains to specific compounds using impregnated paper disks and agar plates. In this study, sterile 6 mm paper disks (Oxoid, Fisher Scientific) were loaded with each herbicidal formulation and deposited on a TSB agar plate 5 days post inoculation. Prior to use on live cultures, optimization of the disk loading volume was required. This was accomplished by delivering 5 different volumes of water (5, 10, 20, 30, and 40 µL) to 5 paper disks in each volume cohort. After drying under ambient temperature in a closed petri dish (150 × 60 mm) for 60 min, the paper disks were examined for adherence to the surface of a tryptic soy agar (TSA) plate. It was determined that the optimal fluid loading volume was 20 µL.

Final preparations were filter sterilized (0.22 μm; Fisher Scientific) under vacuum in 50 mL fractions. The concentration of final solutions was achieved by serial dilution in 2 mL fractions using sterile Milli-Q water (18.2 MΩ-cm) as diluent. Final concentrations chosen were designed to approximate glyphosate exposure in field application conditions, based on the recommended application rate for soybean in Canada and assuming a successful stand rate of 135,000 plants per acre (OMAFRA). Glyphosate resistant (GR) soybean can normally tolerate elevated glyphosate concentrations of 1.35 L/acre (3.33 L/ha). Both acquired glyphosate preparations indicate 540 g of acid equivalent per litre of solution. Following the recommended 10-gallon (37.85 L) water dilution for field spray operations would bring the final tank concentration to 19.22 g of glyphosate per litre. Based on the application rate and stand density of soybean per acre, the maximum expected concentration of glyphosate to reach each plant would be 5.4 mg (0.28 mL/plant tank mix). Herbicide timing for soybean is 24–30 days after planting, approximately at the third vegetative stage of growth (V_3_). Due to the relatively low amount of foliage at the time of herbicide application, a conservative estimate of 50% canopy coverage was used. This assessment further reduces the estimated glyphosate concentration to 2.7 mg per plant, and approximately 40–340 µg of glyphosate per leaf, assuming successful development of the unifoliate leaves, and the first set of trifoliate leaves. A similar quantitative field assessment performed by Harvey & Crothers (1988) found the deposition of glyphosate spray on flax (*Linum usitatissimum*) is largely dependent on the density of planting in the field, as well as equipment settings. Depositions were found to range between 400 and 600 µg per plant, with a minimum exposure of approximately 40 µg per plant required to cause desiccation of the target [70].

Sterile Milli-Q water was used to load a control disk. Preparation of paper disks, including serial dilution of glyphosate products, and loading of each disk across all 5 treatment cohorts were carried out aseptically. Loading solutions were prepared by serial dilution and 20 µL of each solution was transferred to paper disks using a filtered pipette, achieving four different concentrations of glyphosate: 380, 190, 95, and 48 µg. Each petri dish was covered and allowed to dry for 1 h.

### Modified kirby bauer sensitivity assay

Since use of a nutrient-minimum agar (DSMZ 125) reduced the density and uniformity of *Methylobacterium*, a nutrient-rich TSA was used for Kirby Bauer sensitivity assays. Petri dishes containing uniform volumes of 20 mL TSA were divided into quadrants using permanent marker on the outer surface of the base. 100 µL of a 5-day old (∼ 10^8^ CFU/mL) liquid culture (DSM 125) was deposited onto the surface of each separate test plate. The inoculum was evenly spread across the surface of the agar using an L-shaped spreader, then covered and allowed to dry for 1 h in a BSC in darkness at approximately 20^o^C.

Using sterile forceps, dry paper disks loaded with each agrichemical (prepared earlier) were transferred to the test plates, in the center of the quadrant corresponding to the respective disk concentration. A paper disk loaded with sterile water was placed at the center of each petri dish, at the intersection of quadrant lines as a control. Plates were sealed with parafilm and stored inverted. The zone of inhibition surrounding each disk was assessed after 7 days of incubation at 27^o^C in darkness. Zone of inhibition was determined by measuring the diameter of the region with observable absence of microbial growth, inclusive of the paper disk diameter.

### Assessment of *Methylobacterium* sensitivity to glyphosate

Sensitivity of *Methylobacterium* spp. to the commercial formulations (*WeatherMax*® and *Transorb*®) was evaluated at two concentrations of the active ingredient (AI) glyphosate: 0.05% (0.5 mg/mL), and 0.1% (1.0 mg/mL) in 6-well plates (Fisherbrand Cat.No. FB012927); 2 wells contained the *WeatherMax*® product (0.05% and 0.1% glyphosate, respectively), 2 wells contained the *Transorb*® product (0.05% and 0.1% glyphosate, respectively), and the final 2 wells were used as a TSB growth media control. The maximum fill volume for each well across each multi-well plate was set at 5mL to prevent overflow between wells.

To each well, 250µL of a 5-day old liquid culture (DSM 125) was introduced (∼ 10^8^ CFU/mL) and the nutrient content of wells containing TSB only, were normalized by adding sterile water (Milli-Q, 18.2 MΩ-cm). After inoculation, parafilm was used along the perimeter of each multi-well plate to seal the lid to the base and prevent evaporation and contamination. Plates were then placed on a shaker table (Thermo Fisher MaxQ) and rotated at 80 RPM and 27^o^C for 6 days.

Following the incubation period, 100 µL of culture was withdrawn from each well and transferred to 6 corresponding microfuge vials (2 mL) containing 900 µL of sterile isotonic saline solution (0.9% NaCl). The 1:10 mixes were resuspended using a sterile pipette, and then 50 µL was transferred to a TSA plate (20 mL). A sterile L-shaped spreader was used to disperse inoculant across the agar surface, and each plate was sealed using parafilm and incubated at 27^o^C for 7 days inverted. Afterwards, plates were evaluated for colony growth and scored according to a custom scale based on the presentation of colony forming units (CFU): (–) no CFU, (+) if < 30 CFU, (+ +) if > 30 CFU or partial lawn, and (+ + +) if complete lawn present and CFU count impossible.

To assess culture viability following exposure to pure forms of glyphosate, an identical set of replicates were carried out, also in 6-well plates (Fisherbrand Cat.No. FB012927); 2 wells contained pure glyphosate only (0.05% and 0.1%, respectively), 2 wells contained glyphosate at 0.05% and 0.1% with Tween20 (polysorbate-20) at 2% (v/v) in each, 1 well contained Tween20 (2% v/v), and the final well contained only TSB growth media (with nutrient content normalized using sterile water).

### Determination of influence of membrane permeability on cytotoxicity

Determination of the impact of membrane permeability on growth rate was carried out using 3 strains of *Methylobacterium* (*M. organophilum* [NBRC 103119], *M. gnaphali* [NBRC 107716], and *M. jeotgali* [LMG 23639]). The species were selected based on differences in (a) their source of isolation, (b) morphology and pigmentation, (c) rate of proliferation, and (d) strain-specific outcome of the modified Kirby Bauer sensitivity assay using commercial GBH’s.

*Methylobacterium* strains were cultured in 18 mL of TSB media in 50 mL conical tubes (FroggaBio; Cat.No. TL50-500B) after inoculation with 1 mL of a 5-day old 50 mL culture (∼ 10^8^ CFU/mL) grown previously in a 250 mL Erlenmeyer flask (TSB media). The volume in each conical tube was made up to 20 mL through the addition of 1 mL of various stock solutions to achieve the following final conditions: (a) *WeatherMax*® at 0.1% glyphosate, (b) *Transorb*® at 0.1% glyphosate, (c) pure glyphosate at 0.1%, and (d) pure glyphosate at 0.1% with Tween20 at 2% (v/v). Conical tubes were sealed with parafilm, stored horizontally on a tilt table (VWR Rocking Platform; Model 100), and incubated at 27^o^C for 7 days in darkness.

Due to the well-characterized propensity for several species of the *Methylobacterium* to form aggregates in solution after excessively long growth periods or when exposed to stress conditions [71], determination of cell density through optical density (OD) or direct cell counting with a haemocytometer would become unreliable. Therefore, microbial growth rate under each condition was assessed by measurement of pellet dry weight. After 7 days incubation, each conical tube was subjected to centrifugation at 4,700 xg (Thermo Scientific, Sorvall ST16) for 20 min. The supernatant of each tube was removed and discarded. The remaining pellet was then transferred to pre-weighed 2 mL microfuge tubes using isotonic solution (0.9% NaCl) and centrifuged for 10 min at 11,180 xg. The supernatant in each tube was removed and the pellet was freeze dried for 24 h (Labcono Model: 7753020 at -56^o^C and 0.28 mBar). Each tube was subsequently reweighed (Sartorius Practum® 224) to determine the dry weight of each pellet.

To further investigate the influence of Tween20 (polysorbate-20) on the sensitivity of *Methylobacterium* to glyphosate, the aforementioned experiment was carried out again in triplicate, with expanded treatment conditions which included a range of different surfactant dosages with a fixed concentration of pure glyphosate: (a) 0.5-4% (v/v) Tween20 in TSB, and (b) 0.5-4% (v/v) Tween20 with glyphosate 0.1% (w/v) in TSB. Student’s t-test was used to assess differences in dry pellet weight under each growth condition.

### Extraction of intracellular glyphosate and secondary metabolites after RoundUp^®^ exposure

*Methylobacterium* strains (*M. organophilum* [NBRC 103119], *M. gnaphali* [NBRC 107716], and *M. jeotgali* [LMG 23639]) were cultured in 18 mL of TSB media inside 50 mL conical tubes (FroggaBio). Each tube was inoculated with 1 mL of a 5-day old 50 mL culture (∼ 10^8^ CFU/mL) grown previously in a 250 mL Erlenmeyer flask (TSB media).

Conical tubes were sealed with parafilm, stored horizontally on a tilt table (VWR Rocking Platform), and incubated at 27^o^C for 4 days in darkness and constant agitation. On the fourth day (96 h elapsed), the liquid volume of the control cohort for each strain was brought up to 20 mL using sterile water (Ultrapure Milli-Q: Merck Millipore, Toronto, Canada), while the treatment group received a dose of the *Transorb*® formulation (filter sterilized, 0.22 μm PVDF) from a pre-diluted stock solution to achieve two separate final glyphosate concentrations: (a) 0.05 mg/mL (0.005% w/v), and (b) 0.5 mg/mL (0.05% w/v).

After 6 days, each tube was centrifuged at 4,700 xg for 25 min (Thermo Fisher Scientific; Sorvall ST16). Following centrifugation, the supernatant was discarded, and the pellet of each tube was resuspended with 5mL of isotonic 0.9% NaCl solution to remove remnants of the TSB media containing glyphosate, then centrifuged again at 4,700 xg for an additional 15 min. A repeat of the saline solution wash was conducted. After removal of the second saline wash, the pellet in each conical tube was transferred to 2 mL microfuge tubes using a small amount of fresh saline solution, centrifuged at 11,180 xg for 15 min. The supernatant was transferred to a clean microfuge vial and the remaining pellet was flash frozen using liquid nitrogen (LN_2_) and subjected to drying under high vacuum for 24 h (Labcono Model: 7753020 at -56^o^C and 0.28 mBar).

The method of sample preparation was adopted as presented by Li and Kannan (2022) [72] with modifications made for available instrumentation and materials. Briefly, each lyophilized sample was reconstituted with 1.5 mL water:ACN mixture (95:5 *v/v*) containing 0.1% formic acid, and vortexed for 5 min. To tubes containing cell pellets, 2 zirconium oxide grinding beads (5 mm) were added, and vortexed for an additional 5 min 25 MHz using a ball mill (Retsch Mixer Mill MM 400). Cell fragments and particulate matter were settled through centrifugation at 10,000RPM for 10 min. Then 250 µL of supernatant from each tube were loaded onto an Oasis MCX (6 mL carrier, Mississauga, Ontario, Canada) cartridge that had already been preconditioned with 2 mL fractions of methanol and water. Eluent produced from the column were collected immediately as each sample was added to each respective column as only cationic contamination would be absorbed by the MCX cartridge and the target analyte, glyphosate, would flow through unimpeded. The cartridge was washed with an additional 2 mL fraction of water and added to the initial eluent volume. Each collection tube was vortexed for 2 min then 1.5 mL was transferred to a glass vial.

### Liquid chromatography & high resolution tandem mass spectrometry (LC-HRMS/MS)

Identification of glyphosate and target primary metabolites was performed using high performance liquid chromatography-electrospray ionization high-resolution mass spectrometry (HPLC-[ESI]-HRMS). Full scan data was acquired using a QExactive Orbitrap mass spectrometer (Thermo FisherScientific, San Jose CA USA) coupled with a Dionex UltiMate 3000 HPLC (Thermo FisherScientific, San Jose CA USA). A 25 µL of each sample was injected onto a Thermo Scientific Acclaim™ 2.2 μm C18 column (150 mm × 3.0 mm; Canadian Life Science, Peterborough, Canada) using a flow rate of 0.35 mL min^− 1^ with a mobile phase of ultra-pure water (Milli-Q) with 0.1% formic acid (A) and HPLC grade acetonitrile (FisherScientific, Ottawa, Canada) with 0.1% formic acid (B). Mobile phase B was held at 0% for 1.5 min, before increasing to 100% over 4 min. Solvent B was then held at 100% for 3 min before returning to 0% over 2 min, for column re-equilibration. The following conditions were used for heated electrospray ionization (HESI) probe: capillary temperature, 350 °C; sheath gas, 30 arbitrary units; auxiliary gas, 8 arbitrary units; probe heater temperature, 450 °C; S-Lens rf level, 60%; and capillary voltage, 3.9 kV. For HPLC-HRMS, each sample was analyzed in negative mode focusing on a mass range of *m/z* 50 − 700, and data were acquired at 35,000 resolution, with an automatic gain control (AGC) target of 2 × 10^6^, and a maximum injection time (IT) of 540 ms.

For identification, the fragment spectrum of each target compound was obtained using parallel reaction monitoring (PRM). PRM parameters included: automatic gain control (AGC), 2 × 10^5^; maximum injection time (IT), 100 ms; *m/z* 4.0 isolation window, normalized collision energy (NCE) of 30.0 eV, and a focused mass range of *m/z* 50 − 200. All data was analyzed using Thermo Xcalibur (v 3.0.63) software (Thermo Scientific, San Jose, CA, USA), to integrate peak areas.

### Assessment of intracellular formaldehyde

Assessment of intracellular formaldehyde concentration was carried out to determine whether the presence of glyphosate from commercial products would contribute to elevated formaldehyde loads. To examine this, 3 individual strains were cultured in 18 mL of TSB media contained within 50 mL conical tubes (FroggaBio). Each tube was inoculated with 1 mL of a 5-day old 50 mL culture (∼ 10^8^ CFU/mL) contained in a 250 mL Erlenmeyer flask, also in TSB media. A total of 4 conical flasks were inoculated per strain and incubated for 4 days at 27^o^C under constant agitation (VWR Rocking Platform; Model 100). On the fourth day, one of the conical tubes received 1 mL of sterile water (BPure,18 MΩ), while the remaining tubes received a 1 mL aliquot from a series of prepared stocks of the *Transorb*® formulation (filter sterilized, 0.22 μm PVDF) to achieve three final glyphosate concentrations: 50, 250, and 500 µg/mL. After 48-hours, all the conical tubes were centrifuged at 4,700 xg for 20 min. Following centrifugation, the supernatant was discarded, and the pellet of each tube was resuspended in 5 mL of an isotonic solution (0.9% NaCl). After vortex mixing for 1 min, the resuspended cells were subjected to centrifugation at 4,700 xg for 10 min. The supernatant was again discarded, and the pellet was transferred to a 2 mL microfuge tube using 1.5 mL of fresh isotonic saline solution. Each microfuge tube was subsequently subjected to centrifugation at 11,180 xg for 5 min. The supernatant was discarded, and a fresh 1.5 mL fraction of sterile water (BPure, 18 MΩn, non-isotonic) was added to each tube. Two zirconium oxide grinding beads (5 mm) were added to each tube, and mechanical lysis was achieved through grinding for 10 min using a ball mill (Retsch Mixer Mill MM 400) at high speed (25 MHz). Cell fragments and particulate matter were settled through centrifugation at 10,000RPM for 10 min and the supernatant was collected in a clean glass test tube. The pellet was resuspended in an additional 1 mL of sterile water and subjected to a second iteration of mechanical disruption for 5 min. This process was repeated until the total volume transferred to the glass tube was 4.5 mL.

The presence of formaldehyde in spent media and cell pellet extract was determined by dispersive liquid-liquid microextraction ultraviolet visible light spectroscopy (DLLME-UV-Vis) according to Nassiri et al., with minor alterations made to accommodate for reduced sample volume [73]. Briefly, acetyl acetone (2,4-pentanedione) and ammonium acetate were added to each solution type to reach final concentrations of 0.2 mol/L. Glass tubes were inverted to facilitate mixing, and subsequently placed into a hot water bath at 70^o^C for 12 min. After incubation, tubes were allowed to stabilize at room temperature, undisturbed for 15 min. Then 500 µL of anhydrous ethanol and 300 µL of HPLC grade chloroform was added to each glass tube. Each tube was agitated violently to produce a turbid solution as the immiscible aqueous and organic fractions suspended. Separation was subsequently facilitated by moderate centrifugation at 3,000 RPM for 5 min (Thermo Fisher Scientific; Sorvall ST16). Then, 300 µL of the organic fraction was removed and dispensed into a quartz microcell with 300 µL of chloroform diluent, and absorbance was measured at 412 nm promptly.

Evaluation of the DLLME-UV-Vis method showed good linearity in a 5-point calibration curve between 1 and 500 µg/L of formaldehyde spiked in ultrapure water (BPure). All results were normalized against media blank to account for trace formaldehyde in domestic water supply.

### Confirmation of Potency

To prevent contamination of cell cultures, stock solutions containing glyphosate were filtered using 0.2 μm PVDF vacuum-assisted filtration system. While the filter pore size was not anticipated to interfere with the concentration of glyphosate present in final solutions, retention of glyphosate due to interaction with the filter material was unknown and previously untested. At low concentrations of glyphosate (< 1% v/v), filter binding could potentially cause loss of the herbicidal agent.

Twelve soybean plants (*Glycine max*) [non-GMO, Canada domestic white hilum variety] were cultivated from seed and allowed to mature to approximately the unifoliate stage (V_C_). Plants were then transplanted into four 10” pots containing approximately 2 L of soil (Miracle-Gro™ potting mix, 0.21-0.11-0.16) and positioned approximately 2” apart. Plants were allowed to mature to the second trifoliate stage (V_2_). Solutions containing 0.1% glyphosate (1 mg/mL) were then filter sterilized (0.2 μm, PVDF) and placed into 20 mL cosmetic spray bottles and dyed with non-toxic blue food colouring so that leaf saturation would be evident, and drift of any particles that did not contact the plant surfaces would be observable on contrasting surfaces.

## Results

### Sensitivity of *Methylobacterium* to commercial GBH’s

**Fig. 2:**
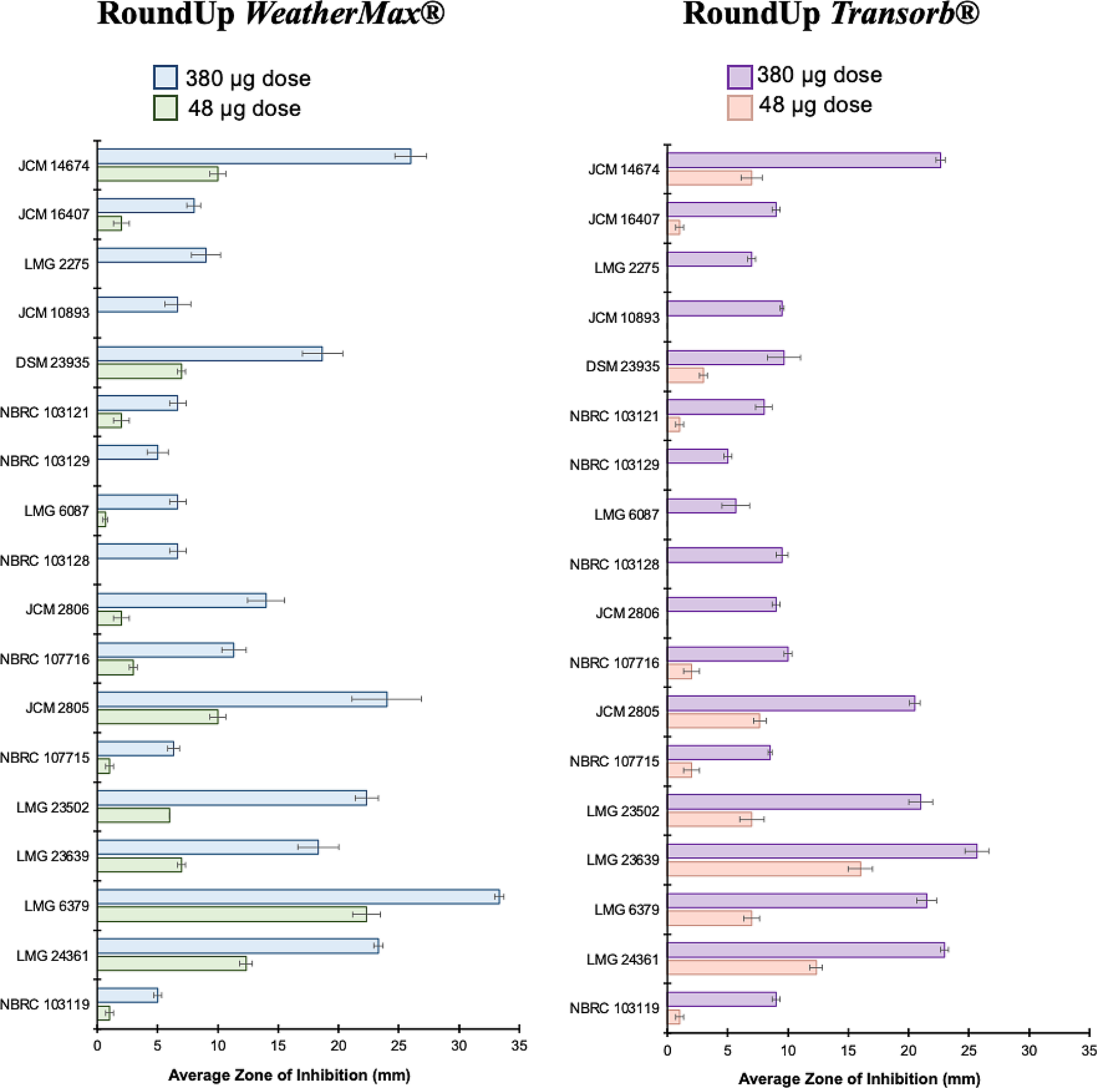
Average (*n* = 3) zone of inhibition of each tested strain of *Methylobacterium* spp. (Table 1) against maximum and minimum concentrations glyphosate (380 µg and 95 µg, respectively) in the *WeatherMax*® [left] and the *Transorb*® [right] products tested

In nearly all cases, sensitivity to both the *WeatherMax*® and *Transorb*® formulations were observed in the Kirby Bauer assay when *Methylobacterium* strains were exposed to disks containing final doses of 95 µg glyphosate or higher. In this work, sensitivity to commercial formulations is defined as the formation of any zone of inhibition greater than the diameter of the paper disks (6 mm), subdivided into three levels; low (1 < > 9 mm), medium (10 < > 19 mm), and high (> 20 mm). Nearly always, a dose-dependent relationship was observed in relation to the zone of inhibition surrounding each paper disk. *M. radiotolerans* (LMG 6379) showed the highest degree of sensitivity to the *WeatherMax*® formulation, even at the lowest dose of 48 µg, and complete clearance of the quadrant at the maximum dose of 380 µg (Fig. 2). Trends between the magnitude of sensitivity and the isolation source of *Methylobacterium* or library of origin could not be established. Notably however, several strains were observed to have tolerance (< 9 mm inhibition zone) to both the *WeatherMax*® and *Transorb*® formulations even at the highest dose administered (Fig. 2); notably, *M. extorquens* (NBRC 103129), *M. organophilum* (NBRC 103119, NBRC 103121), *M. thiocyanatum* (NBRC 103128, JCM 10893), and *M. zatmanii* (LMG 6087). Interestingly however, several strains appeared to have increased sensitivity (> 5 mm inhibition zone) to the *Transorb*® formulation compared to identical experiments involving the *WeatherMax*® solution (NBRC 103119, LMG 23639), while others had greater sensitivity to the latter (DSM 23935, JCM 2806). As each formulation contains a proprietary blend of surfactants, the type and total surfactant content of either product cannot be used as a basis for the observed sensitivity.

**Fig. 3:**
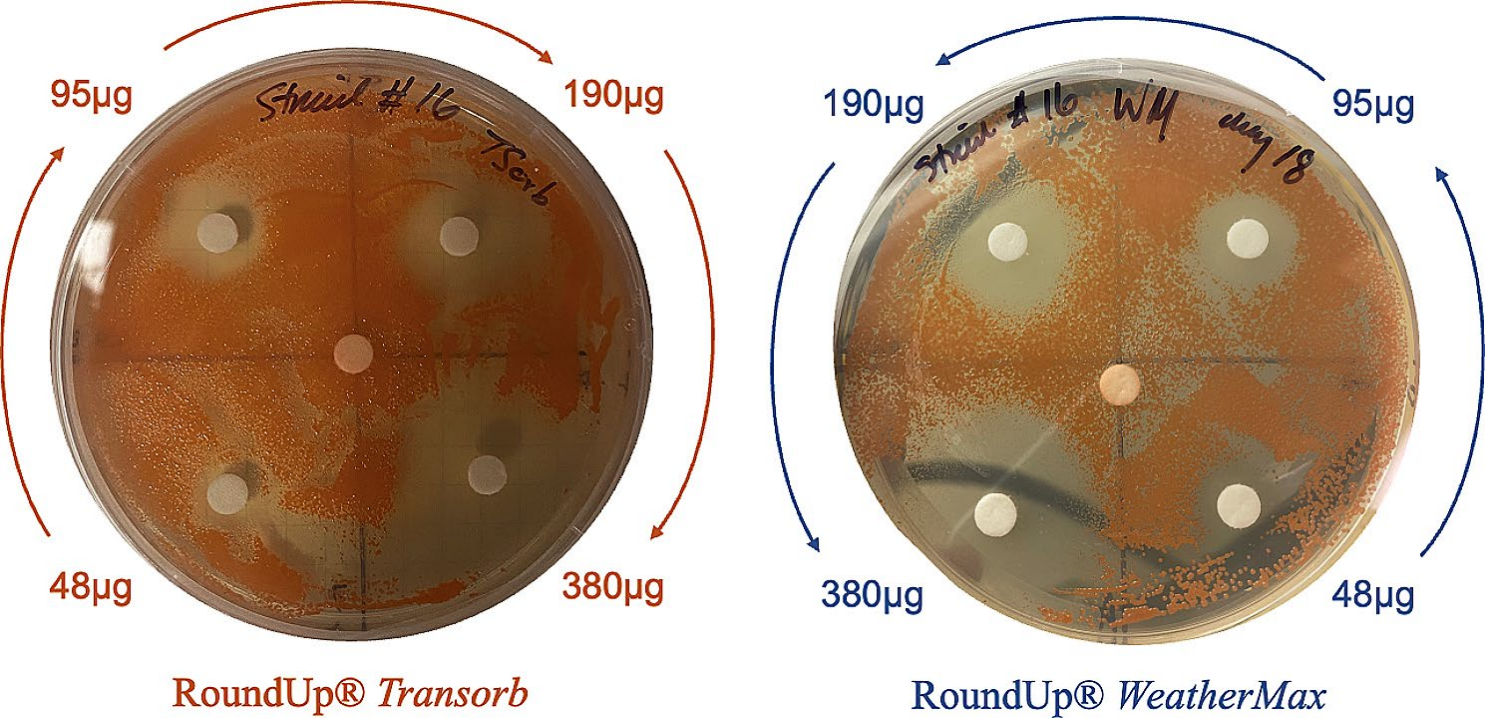
Representative photograph illustrating zone of inhibition of *Methylobacterium gnaphali* (NBRC 107716) to four concentrations of *Transorb*® [left] and *WeatherMax*® [right]

Replication of the modified Kirby Bauer method was conducted using pure formulations of glyphosate in the range of 48–380 µg. Across all 18 strains of *Methylobacterium*, no measurable zone of inhibition was detected and, in several instances, *Methylobacterium* colonized glyphosate-impregnated disks. This apparent insensitivity to glyphosate in its pure form was consistent across all 3 replicates of the experiment. Sterile control disks, loaded with deionized water and included at the center of each test plate showed no indication of sensitivity in any strain, as expected (Fig. 3).

### Assessment of permeability on cell viability

**Fig. 4:**
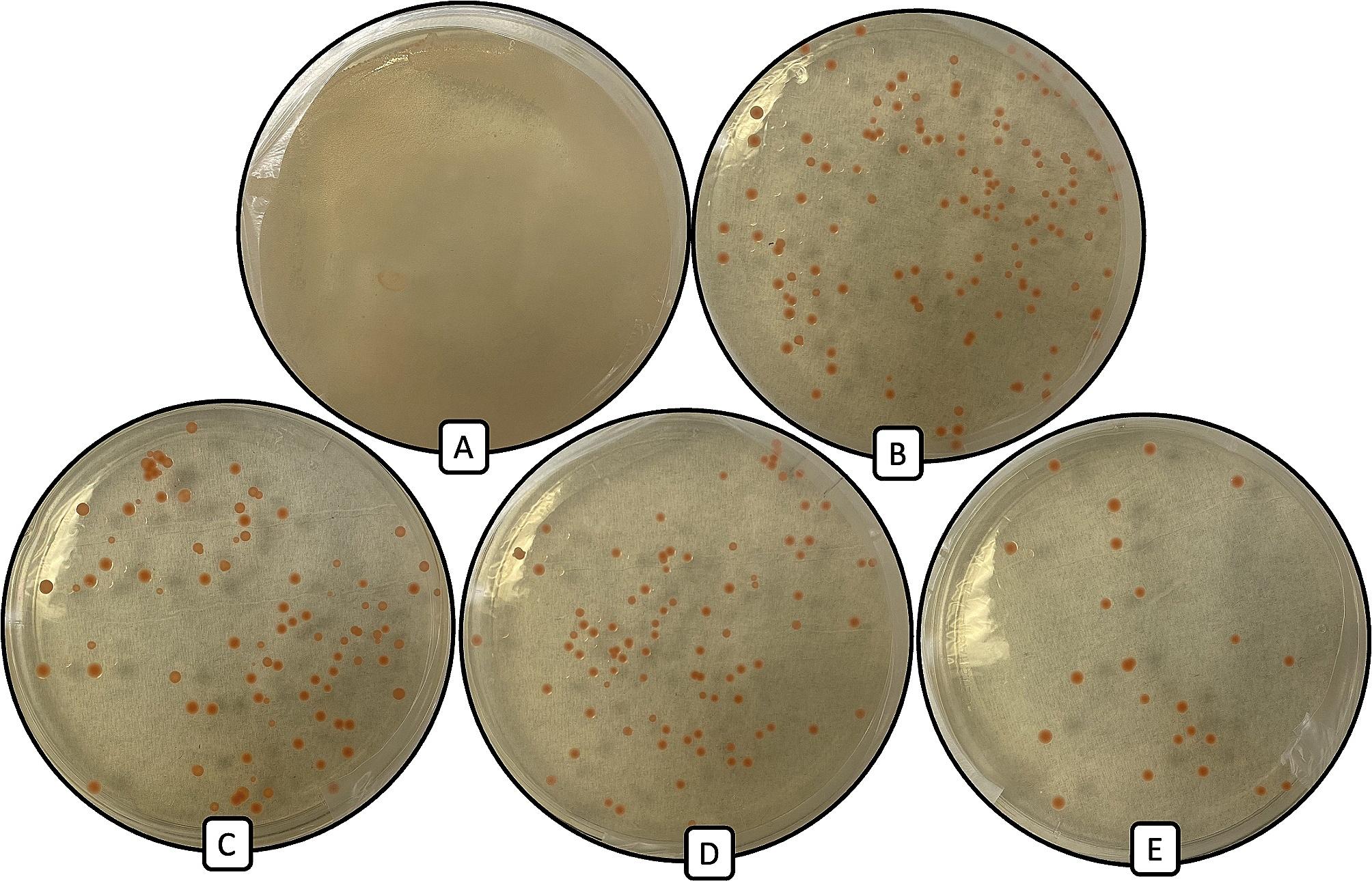
Representative photograph illustrating results of cell viability test from cultures containing 0.1% pure glyphosate (1 mg/mL) with *Methylobacterium gnaphali* (NBRC 107716) with varying concentrations of Tween20 (polysorbate-20); (**A**) control, (**B**) 0.5%, (**C**) 1.0%, (**D**) 2.0%, (**E**) 4.0% (v/v). Frame (**A**) depicts confluent growth of NBRC 107716

In experiments involving 6-well plates which used the *WeatherMax*® and *Transorb*® final formulations as the source of glyphosate (0.5 and 1.0 mg/mL glyphosate), culture growth was absent even after incubation for more than 7 days, and a measurable pellet could not be obtained. To discern whether the GBH formulations were lethal or bacteriostatic towards *Methylobacterium*, aliquots of 500 µL from each treatment were transferred to separate flasks of fresh media (50 mL TSB in 250 mL Erlenmeyer) and incubated for a further 7 days at 27^o^C. In each case where inoculum had previously been exposed to commercial GBH’s, growth in fresh media was not detectible. This phenomenon was further illustrated during viability tests where strains were unable to recover activity after transfer of a 50 µL aliquot from wells spiked with GBH’s to GBH-free TSA (Table 2). Direct exposure of *Escherichia coli* to the same concentrations of both the *Transorb*® and *WeatherMax*® GBH’s, did not impact culture recovery and proliferation of the microorganism, producing a confluent GBH-free TSB plate each time. Interestingly, wells containing pure glyphosate at the same concentration (0.5 and 1.0 mg/mL) did produce viable cells that proliferated normally while in media spiked with glyphosate, and when transferred to glyphosate-free growth media (Table 3; Fig. 4).

**Table 2:** Cell viability test (*n* = 1) of each strain of *Methylobacterium* spp. against two concentrations of the *Transorb*® (Tsorb) and *WeatherMax*® (WMax) product formulations. Negative control involved nutrient-rich trypic soy broth (TSB) with no addition of commercial products

Species	Strain	Cell Viability				
		Neg. Control	Tsorb 0.05%	Tsorb 0.1%	WMax 0.05%	WMax 0.1%
M. *extoquens*	JCM 2805	+ + +	−	−	−	−
	JCM 2806	+ + +	−	−	−	−
	NBRC 103129	+ + +	−	−	−	−
M. *gnaphali*	NBRC 107716	+ + +	−	−	−	−
M. *iners*	JCM 16407	+ + +	−	−	−	−
M. *jeotgali*	LMG 23639	+ + +	−	−	−	−
M. *organophilum*	NBRC 103119	+ + +	−	−	−	−
	NBRC 103121	+ + +	−	−	−	−
M. *oryzae*	LMG 23502	+ + +	−	−	−	−
M. *oxalidis*	NBRC 107715	+ + +	−	−	−	−
M. *phylospharae*	LMG 24361	+ + +	−	−	−	−
M. *radiotolerans*	LMG 6379	+ + +	−	−	−	−
M. *rhodinum*	LMG 2275	+ + +	−	−	−	−
M. *thiocyanatum*	NBRC 103128	+ + +	−	−	−	−
	JCM 10893	+ + +	−	−	−	−
M. *zatmanii*	LMG 6087	+ + +	−	−	−	−
*Methylobacterium spp*.	DSM 23935	+ + +	−	−	−	−
	JCM 14674	+ + +	−	−	−	−
E. *coli*	NM 522	+ + +	+ + +	+ + +	+ + +	+ + +

(−) no CFU, (+) if < 30 CFU, (+ +) if > 30 CFU or partial lawn, and (+ + +) if complete lawn present and CFU count impossible

**Table 3:** Cell viability test (*n* = 2) of each strain of *Methylobacterium* spp. against two concentrations of pure glyphosate (GLY), with and without Tween20 (polysorbate-20). Negative control involved nutrient-rich trypic soy broth (TSB) with no addition of glyphosate or Tween20

Species	Strain	Cell Viability					
		Neg. Control	2% Tween20	GLY 0.05%	GLY 0.1%	GLY 0.05% + 2% Twen20	GLY 0.1% + 2% Twen20
M. *extoquens*	JCM 2805	+ + +	+ +	+ + +	+ + +	+ +	+
	JCM 2806	+ + +	+ +	+ + +	+ + +	+ +	−
	NBRC 103129	+ + +	+ +	+ + +	+ + +	+ +	+
M. *gnaphali*	NBRC 107716	+ + +	+ +	+ + +	+ + +	+ +	+
M. *iners*	JCM 16407	+ + +	+ +	+ + +	+ + +	+ +	+
M. *jeotgali*	LMG 23639	+ + +	+ +	+ + +	+ + +	+ +	−
M. *organophilum*	NBRC 103119	+ + +	+ +	+ + +	+ + +	+	+
	NBRC 103121	+ + +	+ +	+ + +	+ + +	+ +	+
M. *oryzae*	LMG 23502	+ + +	+ +	+ + +	+ + +	+ +	−
M. *oxalidis*	NBRC 107715	+ + +	+ +	+ + +	+ + +	+	+
M. *phylospharae*	LMG 24361	+ + +	+ +	+ + +	+ + +	+ +	−
M. *radiotolerans*	LMG 6379	+ + +	+ +	+ + +	+ + +	+ +	−
M. *rhodinum*	LMG 2275	+ + +	+ +	+ + +	+ + +	+ +	+
M. *thiocyanatum*	NBRC 103128	+ + +	+ +	+ + +	+ + +	+ +	+
	JCM 10893	+ + +	+ +	+ + +	+ + +	+ +	+
M. *zatmanii*	LMG 6087	+ + +	+ +	+ + +	+ + +	+ +	−
*Methylobacterium spp*.	DSM 23935	+ + +	+ +	+ + +	+ + +	+ +	−
	JCM 14674	+ + +	+ +	+ + +	+ + +	+ +	−
E. *coli*	NM 522	+ + +	+ +	+ + +	+ + +	+ + +	+ + +

(−) no CFU, (+) if < 30 CFU, (+ +) if > 30 CFU or partial lawn, and (+ + +) if complete lawn present and CFU count impossible

However, in the second iteration of the experiment which introduced custom mixes of pure glyphosate and the non-toxic surfactant Tween20 (polysorbate-20), deleterious effects to growth rate and viability were observed (Table 3). Specifically, the lowest concentration of Tween20 required to induce negative impacts on growth was 0.5% (v/v). Importantly, the negative effects to *Methylobacterium* viability appeared to be a function of the surfactant concentration. In experiments involving uniform concentrations of pure glyphosate but varying surfactant content, the *Methylobacterium* strains tested, exhibited slower growth and a marked reduction in the number of viable colonies on recovery plates after 7 days in response to increasing surfactant (Tween20) concentrations (Fig. 4).

**Fig. 5:**
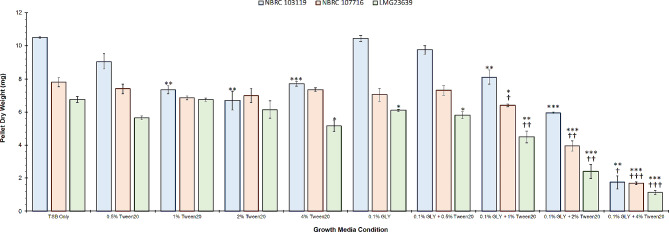
Graphical representation of dry pellet weight of three distinct *Methylobacterium* strains when cultured in tryptic soy broth (TSB) containing a fixed quantity of pure glyphosate (0.1% w/v) in relation to changes in the presence of Tween20 (polysorbate-20), relative to controls containing Tween20 alone (*n* = 4). The Student’s t-test was used to assess statistical difference between groups. A star (*) indicates statistical difference in pellet weight between control conditions (TSB only) and following the application of a treatment (*p* < 0.05 = *, *p* < 0.01 = **, *p* < 0.001 = ***). A dagger (†) indicates statistical difference comparing pellet weight between the application of Tween20 and the corresponding application of Tween20 with the addition of glyphosate (*p* < 0.05 = †, *p* < 0.01 = ††, *p* < 0.001 = †††)

In culture growth experiments with three distinct *Methylobacterium* strains, conical tubes containing nutrient rich TSB media spiked with 0.1% pure glyphosate (1.0 mg/mL) resulted in statistical decreases in pellet weight only seen in M. *jeotgali* (LMG 23639). However, when the surfactant Tween20 is added to the growth media in combination with glyphosate, final pellet weights after 6 days were markedly reduced relative to controls despite receiving uniform inoculant (1 mL of 10^8^ CFU/mL). While the pellet weight of M. *organophilum* (NBRC 103119) was significantly lower in the presence of Tween20 at 4% compared to TSB alone (7.70 ± 0.14 mg and 10.50 ± 0.07 mg, respectively), concomitant exposure of M. *organophilum* to glyphosate (1.0 mg/mL) and Tween20 also at 4%, caused significant decrease in pellet weight, especially when compared to glyphosate alone (1.83 ± 0.32 mg and 10.30 ± 0.13 mg, respectively, *p* ≅ 0.0002) (Fig. 5). Over a 60% reduction in biomass was also observed in cultures of M. *gnaphali* (NBRC 17716) and M. *jeotgali* (LMG 23639) when exposed to glyphosate and a surfactant compared to glyphosate alone (Fig. 5). In fact, statistical decreases in pellet weight were observed across all three strains of *Methylobacterium* grown in media with glyphosate and Tween20 at 4% when compared to TSB only and exclusively Tween20 at 4% (Fig. 5).

Importantly, conical tubes spiked with either the *WeatherMax*® or *Transorb*® formulations at identical glyphosate concentrations to pure solutions tested (1.0 mg/mL and 0.5 mg/mL glyphosate) failed to produce any measurable biomass in any of the tested strains, in triplicate. Every attempt to inoculate media with *Methylobacterium* which already contained the *WeatherMax*® or *Transorb*® formulations failed to produce viable cultures, even when incubated beyond 12 days.

### Assessment of intracellular formaldehyde

**Fig. 6:**
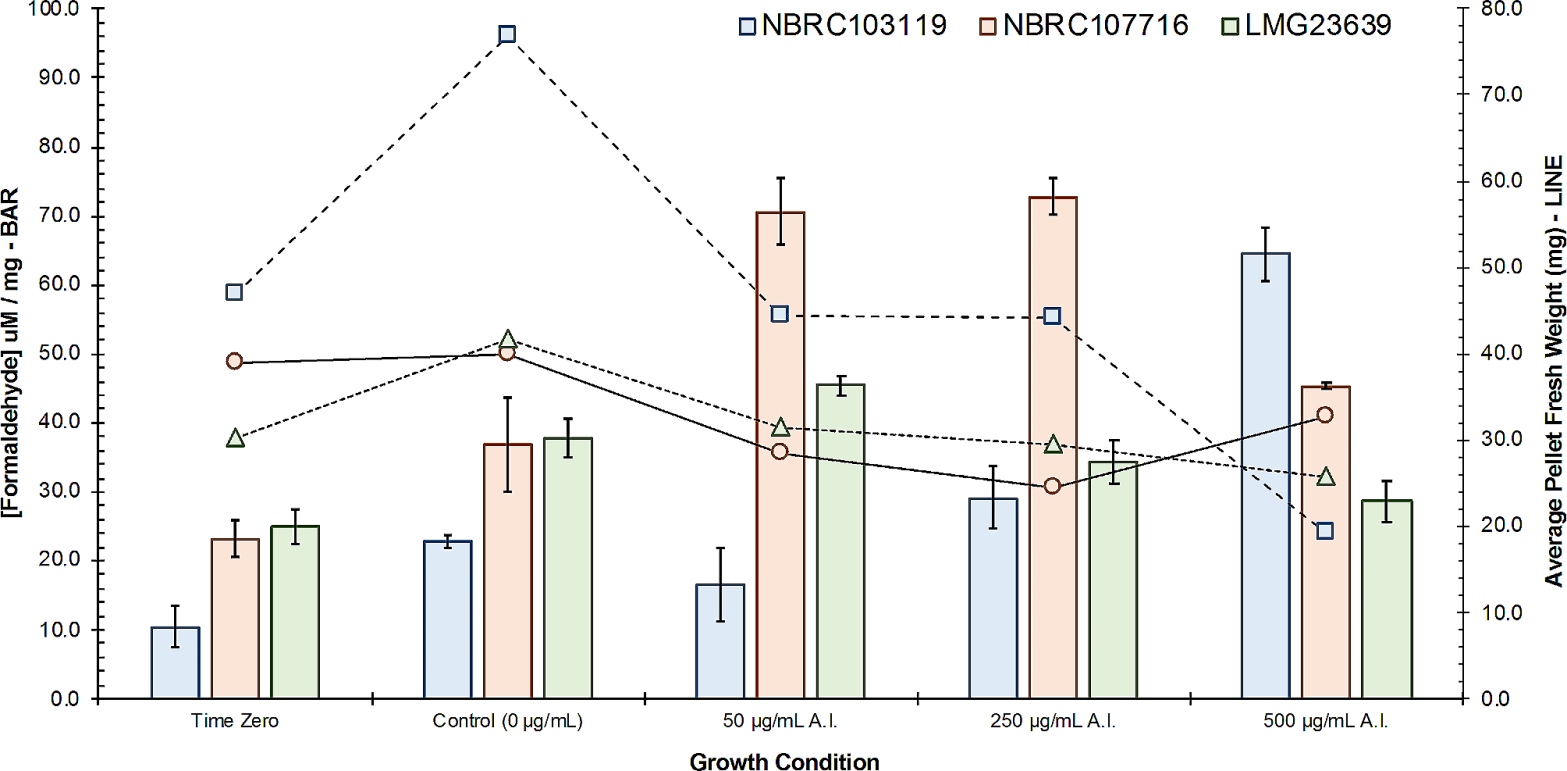
Graphical representation of average pellet fresh weight (line, right axis) of three distinct *Methylobacterium* strains when cultured in tryptic soy broth (TSB) containing fixed quantities of the active ingredient (AI), glyphosate, obtained from the *Transorb*® commercial product, and corresponding intracellular formaldehyde concentrations (bar, left axis) after 4 days of growth at 27^o^C (*n* = 4)

In experiments evaluating the intracellular concentration of formaldehyde, three morphologically distinct strains of *Methylobacterium* were included: *M. organophilum* (NBRC 103119), *M. gnaphali* (NBRC 107716), and *M. jeotgali* (LMG 23639). In all cases, increasing concentrations of glyphosate from the *Transorb*® formulation exposed to growing cultures, resulted in greater quantities of intracellular formaldehyde detectable by the DLLME-UV-Vis method, relative to controls. Because the spectrophotometric method is destructive, pre-exposure formaldehyde levels could only be obtained from a separate time-zero cohort; after 4 days of growth in TSB, intracellular formaldehyde concentrations for *M. organophilum* (NBRC 103119), *M. gnaphali* (NBRC 107716), and *M. jeotgali* (LMG 23639) were 10.26 ± 3.08 nM/mg (3.7 µg/L), 20.09 ± 4.81 nM/mg (7.2 µg/L), and 20.72 ± 5.00 nM/mg (7.5 µg/L), respectively (Fig. 6). The control cohort, which after 4 days of undisturbed growth were spiked with 1 mL of sterile isotonic solution (0.9% NaCl [w/v]) and were then harvested 2 days later, showed moderate increases to intracellular formaldehyde content; 22.83 ± 1.00 nM/mg (8.2 µg/L), 36.94 ± 6.86 nM/mg (13.3 µg/L), and 37.85 ± 2.78 nM/mg (13.7 µg/L).

Interestingly though, with the addition of the *Transorb*® product (glyphosate at 50 µg/mL) on the fourth day of growth, the average intracellular formaldehyde content of *M. organophilum* decreases approximately 27% (16.54 ± 5.35 nM/mg), while *M. gnaphali* and *M. jeotgali* experience an average increase of 91% (70.58 ± 4.81 nM/mg) and 20% (45.49 ± 1.35 nM/mg) relative to their control cohort, respectively. At 250 µg/mL of glyphosate in the growth media, average intracellular formaldehyde content peaks for *M. gnaphali* at 72.79 ± 2.55 nM/mg, then declines to 45.37 ± 0.47 nM/mg at the maximum exogenous glyphosate application (500 µg/mL). A similar trend is also observable for *M. jeotgali*, where exposure to the two higher glyphosate concentrations resulted in a decrease of average intracellular formaldehyde (34.46 ± 3.14 nM/mg and 28.60 ± 3.47 nM/mg). Only *M. organophilum* exhibited disproportional increases in average intracellular formaldehyde content with high concentrations of exogenous glyphosate (29.15 ± 4.46 nM/mg and 64.50 ± 4.42 nM/mg), suggesting formaldehyde tolerance may have a species-dependant component.

### Measurement of intracellular glyphosate and secondary metabolites

**Fig. 7:**
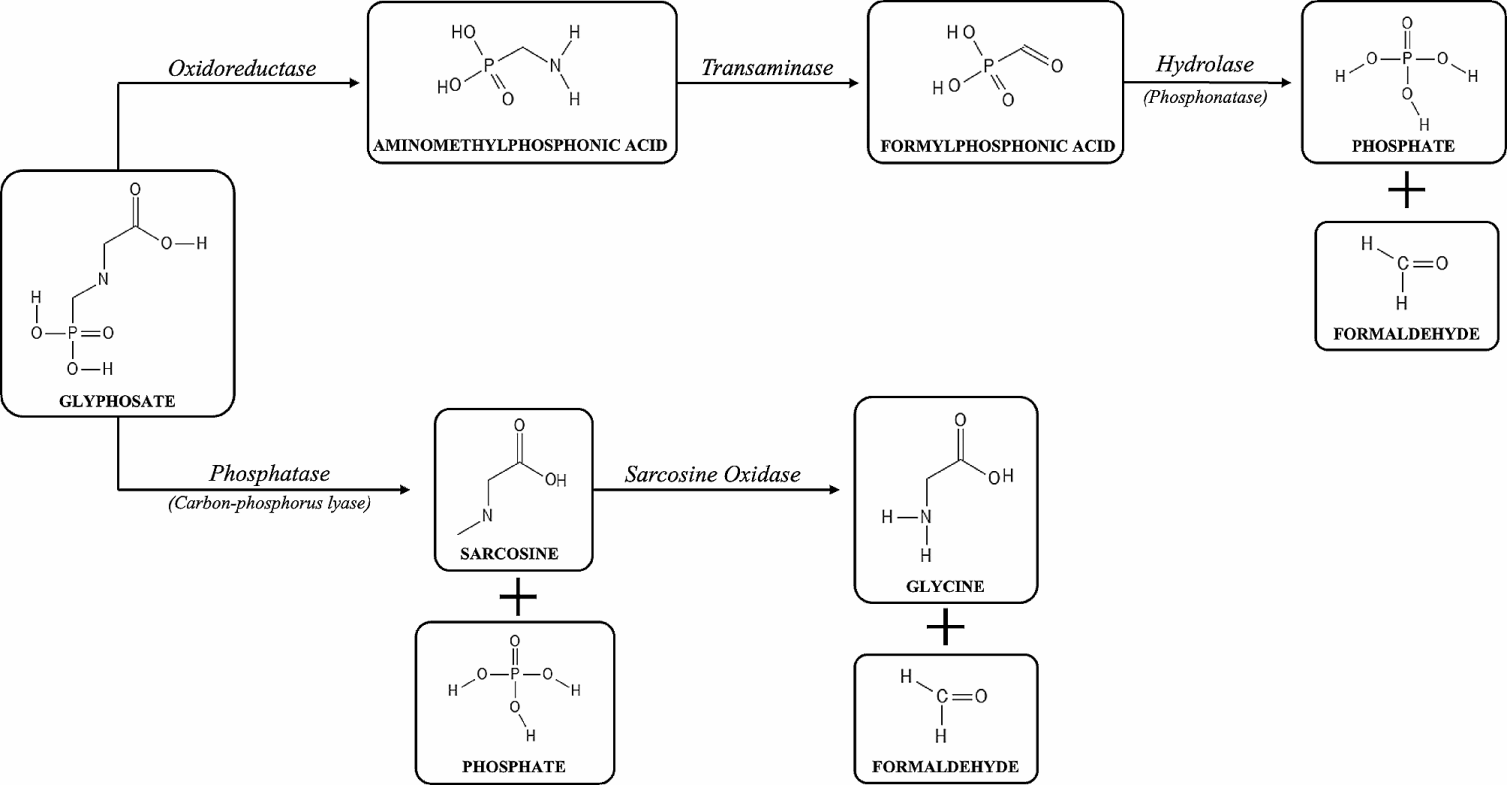
Schematic illustrating basic metabolic pathways that may lead to increased intracellular formaldehyde load in *Methylobacterium* spp

Initially, severe matrix effects interfered with the reliable detection of glyphosate and suspected primary metabolites (AMPA and sarcosine) extracted from cell pellets of *Methylobacterium* exposed to *Transorb*® in the growth media and analyzed using HPLC-[ESI]-HRMS/MS. However, use of mixed-mode strong cation-exchange cartridges (Oasis MCX) as a cleanup method prior to analysis, enabled clear detection of all analytes. Specifically, the peak area of glyphosate detected in cell pellets was approximately 8-fold greater when MCX cartridges were used for the removal of cationic interference in the cell lysate when compared to the use of unprocessed extracts (data not shown). Using the targeted parallel reaction monitoring (PRM) method, fragment ions of the target analyte, glyphosate, and two potential primary metabolites produced by different degradation pathways were used to quantify each compound (Tables 4 and 5). For example, degredation of glyphosate through an oxidoreductase pathway would be expected to yield aminomethylphosphonic acid (AMPA), while degradation via phosphatase would be expected to produce sarcosine as an intermediate metabolite (Fig. 7).

**Table 4:** Glyphosate, target metabolites, and optimized PRM method parameters. Product ions were used for quantification of the compounds by Xcalibur (v. 3.0.63) data processing module (Quan Browser)

Target Analyte	Chemical Formula (M)	Precursor [M-H]^−^ (m/z)	Normalized Collision Energy, eV (NCE)	Product Ions (m/z)
Glyphosate	C_3_H_8_NO_5_P	168.00673	30	78.95902 80.97466 110.00119 124.01685 149.99619
Aminomethylphosphonic Acid (AMPA)	CH_6_NO_3_P	110.00125	30	62.96417 78.95901 80.97469
Sarcosine	C_3_H_7_NO_2_	88.04040	30	60.99348 71.01444 87.00938

**Table 5:** Average intracellular concentration [pMol / mgDW] and standard error of glyphosate and target metabolites in cell pellets of *M. organophilum*, M. *gnaphali*, and *M. jeotgali* determined by UHPLC-[ESI]-HRMS/MS (*n* = 5). Compounds that were unable to be detected are indicated as not-detected (n.d.). A dagger (†) indicates statistical difference comparing concentration of detected compounds between the application of *Transorb*® commercial GBH and identical negative controls (*p* < 0.05 = †)

Target Analyte	*M. organophilum* (NBRC 103119)		*M. gnaphali* (NBRC 107716)		*M. jeotgali* (LMG 23639)	
	*Transorb*® *Media Concentration*					
	*50 µg/mL*	*500 µg/mL*	*50 µg/mL*	*500 µg/mL*	*50 µg/mL*	*500 µg/mL*
Glyphosate	0.23 ± 0.03^†^	2.36 ± 0.01^†^	0.03 ± 0.00	1.32 ± 0.11^†^	0.41 ± 0.02^†^	2.67 ± 0.18^†^
Aminomethylphosphonic Acid (AMPA)	1.18 ± 0.11^†^	13.54 ± 0.45^†^	3.63 ± 0.39^†^	27.09 ± 1.38^†^	1.62 ± 0.21^†^	n.d.
Sarcosine	n.d.	n.d.	n.d.	n.d.	n.d.	n.d.

Despite detection and confirmation of glyphosate and AMPA by comparison of fragmentation patterns against those produced by authentic standards, the well-characterized alternative glyphosate metabolite, sarcosine (N-methyl glycine), could not be detected in any strain, irrespective of *Transorb*® concentration. Interestingly though, method controls of the sarcosine internal standard (STD) were abundantly low compared to glyphosate and AMPA standards, and were particularly masked by matrix effects (NL: 1.0E3) in QA/QC controls. Limit of detection (LOD) for sarcosine (50.0 pMol/L) standards were notably higher than the LOD for authentic standards of glyphosate and AMPA (11.9 and 17.8 pMol/L, respectively), which aligns with the findings of similar studies involving the detection of sarcosine. Although unconfirmed, it is also possible that the detectability of sarcosine may have been impacted by rapid oxidation to form glycine and formaldehyde, relegating the presence of sarcosine in the cytosol to be only transitory.

**Fig. 8:**
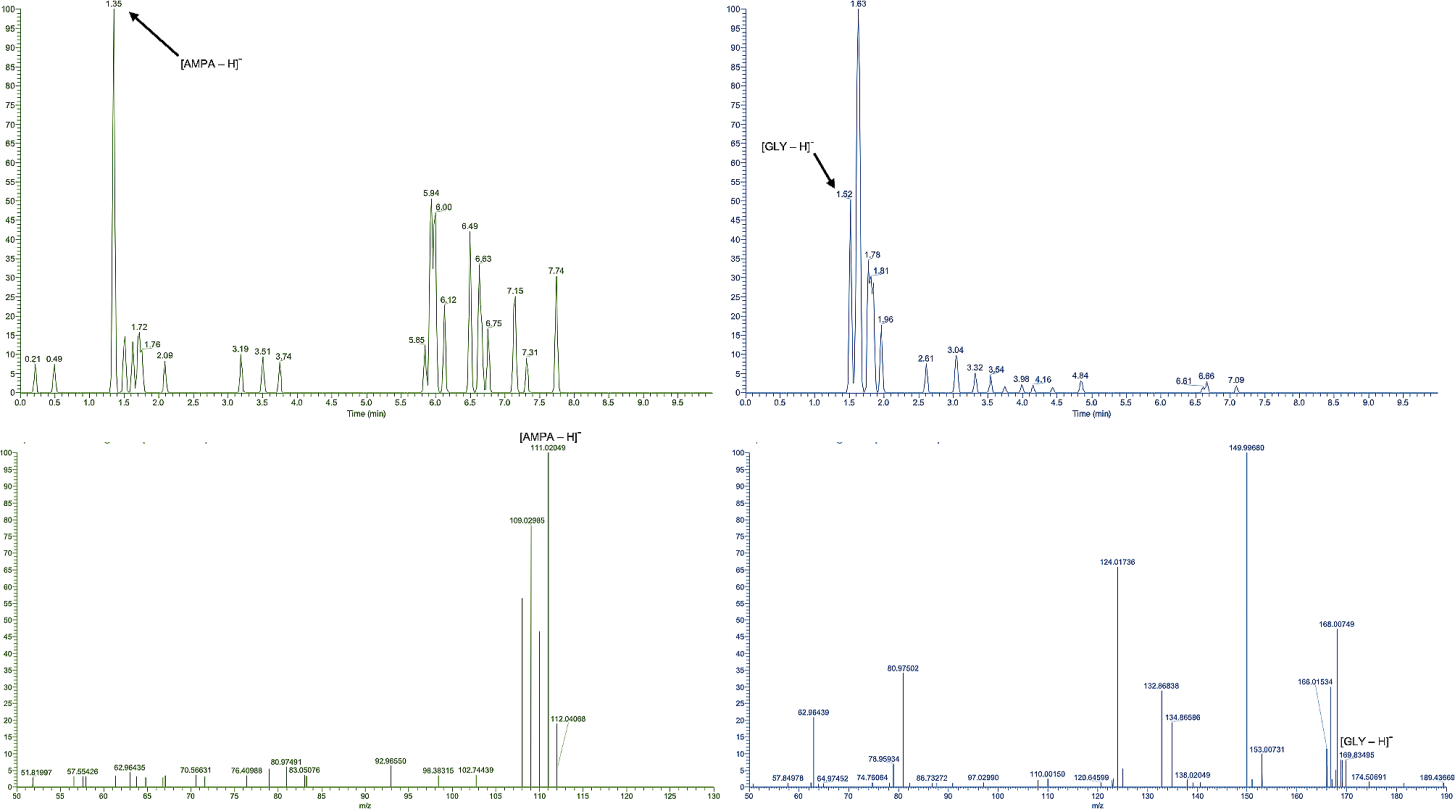
Mass spectrum of target metabolite AMPA and associated chromatogram (left), and glyphosate (right) detected intracellularly from a pellet of *M. gnaphali* (NBRC 107716) after 4 days incubation in TSB spiked with *Transorb*® formulation (500 ug/mL A.I.)

In each of the *Methylobacterium* strains analyzed, a near dose-dependent relationship between glyphosate exposure and the AMPA metabolite was observed. For example, in response to a tenfold increase to glyphosate applied extracellularly in the form of the *Transorb*® commercial product, the average intracellular AMPA concentration in M. *organophilum* (NBRC 103119) rose by 11-fold and M. *gnaphali* by 8-fold (NBRC 107716). The full MS/MS spectra of glyphosate and one of the primary metabolites, AMPA, detected in a cell pellet of *M. gnaphali* (NBRC 107716) are shown in Fig. 8.

These results confirm that exposure of *Methylobacterium* spp. to glyphosate in the *Transorb*® commercial formulation, do result in detectable levels of intracellular glyphosate and one of its known metabolites, AMPA. Cross-analysis of all purified standards did not result in detectable quantities of any other standards, eliminating the possibility of in-bottle degradation as a source of AMPA, and further confirming its presence in samples was as a result of metabolic activity of the *Methylobacterium* spp. tested.

## Discussion

### Glyphosate cytotoxicity in *Methylobacterium*

Investigations of implications of the widespread use and reliance on glyphosate has existed for several decades and has involved contributions from a vast range of disciplines addressing ecological concerns, human health impacts, and the economics of the agri-food industry. However, a more thorough examination of the effects of glyphosate and GBH’s on the phyllosphere have been largely absent in the relevant body of literature.

At present, several species of *Methylobacterium* have been reported to contain the glyphosate sensitive (GS) isoform of EPSP synthase (EC: 2.5.1.19) including M. *aquaticum* [MA-22 A], M. *phyllosphaerae* [CBMB-27], M. *radiotolerans* [JCM 2831], and M. *oryzae* [CBMB-20] [74–76]. While complete genomic sequencing of all known species of *Methylobacterium* has not yet been conducted, the reported presence of a sensitive EPSP synthase in several species is supportive of our findings of sensitivity. Uniquely however, our results suggest that the sensitivity of *Methylobacterium* to glyphosate may be a function of the quantity of glyphosate that is able to enter the cytosol. Across all growth experiments conducted, there was no statistical difference in growth rate or viability of *Methylobacterium* when exposed to pure forms of glyphosate. In experiments where permeability of the lipid bilayer was deliberately increased using Tween20 (polysorbate-20), cytotoxicity increased as a function of both the glyphosate dose and the content of surfactant in the growth media.

Commercial herbicide formulations are known to contain a wide range of additives including surfactants, which aid in product dispersibility and adherence to plant surfaces. GBH’s specifically, often contain 1,4-dioxane as a preservative and polyoxyethyleneamine (POEA) as a surfactant. The toxicity of surfactants including POEA and its structural analogues has been investigated previously, with a majority of reports indicating that any observable toxicity in animals and tissue cultures exposed to GBH’s are likely attributable to the surfactant itself [77, 78].

The use of Tween20 for the adjustment of membrane permeability has been documented extensively [79–85]. As a non-ionic surfactant, Tween20 effectively increases cell permeability without inducing the toxic effects exhibited with other surfactant classes. While the addition of Tween20 to cell cultures in the range of 0.1–10% (v/v) has previously been reported to cause disruptions to growth due to changes in membrane permeability and cell turgidity, our data indicates that *Methylobacterium* tolerate the presence and activity of Tween20 with moderate (< 30%) decreases in biomass relative to controls (Fig. 5). Crucially, examination of the surfactant-only cohort, where growth rate was modestly decreased, revealed the cultures to remain viable beyond 10 days which would otherwise be impossible if the presence of Tween20 induced rupture of the lipid bilayer. Previously, Tween20 was examined in conjunction with glyphosate effectivity on several field-crops - including wheat, barley, oats, and rape - where the addition of Tween20 to tank mixes was found to enhance effectivity of glyphosate [86]. The results of the present work align with existing literature and indicate that the presence of surfactants enhances the effects of glyphosate likely as a result of increasing membrane permeability and enabling greater cytosolic concentrations of glyphosate in bacterial cells.

As a gram-negative bacteria, *Methylobacterium* have hardened defence systems that prevent biomolecules and synthetic chemicals from damaging critical cellular components. Most often, foreign molecules are repelled by the outer membrane (OM) – an asymmetric bilayer that is fully coated with lipopolysaccharides on its outer surface – without ever gaining entry to the cell [87]. Should a foreign molecule pass through the OM by means of a porin protein, a second layer of protection within the periplasmic space (the fluid envelope existing between the outer and inner membranes) would likely activate to further protect the cell. In addition to efflux pumps – which work to move foreign matter back across the OM and into the extracellular matrix – the periplasmic space is known to contain a litany of defensive and detoxifying enzymes including phosphatases, proteases, and endonucleases in gram-negative bacteria [88–90].

In the absence of a surfactant and because of the architecture of the cell wall shared amongst gram-negative bacteria, *Methylobacterium* may simply reject the molecule and process what small quantities of glyphosate pass through porin proteins in the periplasmic space. In all replicates of the modified Kirby Bauer assay, none of the tested strains showed signs of sensitivity to pure glyphosate; however, moderate sensitivity (10 > < 19 mm zone of inhibition) was clearly observable in tests involving commercial formulations containing glyphosate. Similarly, growth media spiked with pure glyphosate showed no statistical impact on growth rate or viability, whereas use of commercial formulations as the source of glyphosate produced non-viable cultures at identical concentrations. In the presence of a control surfactant (Tween20) however, negative effects on *Methylobacterium* growth returned. We postulate that this effect is likely a result of disrupting the integrity of the OM which renders efflux pumps overwhelmed by the rapid ingress of exogenous glyphosate induced by the presence of Tween20. The effect of the surfactant is particularly evident in experiments where the concentration of glyphosate was static, and lower biomass was recovered as a function of increasing the concentration of Tween20 (Fig. 5). In practice, the precise cause for the negative effects of commercial GBH’s on *Methylobacterium* can only be speculated because the proprietary nature of both product formulations tested, prevent direct testing of specific ingredients.

Crucially though, in both the Kirby Bauer assay and cell viability tests, *E. coli* did not indicate sensitivity to GBH’s, mixes of glyphosate and Tween20, or glyphosate alone. This observation contradicts the hypothesis that surfactants enhance glyphosate toxicity in *Methylobacterium* by enabling bypass of cellular infrastructure designed for detoxification leading to a cessation of AAA biosynthesis. While the restoration of AAA biosynthesis through eventual elimination of glyphosate may be remarkably slow in *Methylobacterium*, the loss of culturability of *Methylobacterium*, observed even when transferred to fresh nutrient-rich agar, suggests a potential secondary mechanism through which permanent cell damage may occur.

We therefore suggest that intracellular glyphosate may not only reduce AAA biosynthesis through inhibition of EPSP synthase, but it also might be metabolized to more toxic products including formaldehyde, through alternate pathways. Through review of the metabolic capabilities of several *Methylobacterium* strains, we postulate that intracellular glyphosate may contribute to increases in cytosolic formaldehyde by two candidate pathways: (a) C-N cleavage by oxidoreductase, and/or (b) C-P cleavage by phosphatase (Fig. 7).

### Proposed mechanism of toxicity

Two enzymes previously isolated from *Methylobacterium* spp., carbon-phosphorus lyase (C-P lyase) [76] and sarcosine oxidase (SO) [91, 92], may cause the detected rises in intracellular formaldehyde through a two-step process. Briefly, once inside the cell, glyphosate may initially be cleaved by CP lyase into phosphate and sarcosine, which in turn may be further cleaved into glycine and formaldehyde in a second step by SO (Fig. 7). Criticism for this postulation may stem from the fact that the mere presence of CP lyases do not necessarily imply activity against glyphosate, as a higher degree of substrate specificity for glyphosate degradation has been suggested [93]. Additionally, our work indicates no detectable levels of sarcosine were observed in any of the tested *Methylobacterium* strains, irrespective of glyphosate dose. However, the mechanics and kinetics which underly glyphosate degradation by CP lyases remains poorly understood. Available reports suggest that the ability for some CP lyases to degrade glyphosate may either be a result of isoforms with inherently lower substrate specificity in some organisms, or the presence of glyphosate-like molecular analogues naturally present in the environment which necessitate isoforms of narrow substrate specificity that also happen to be capable of degrading glyphosate [94].

More likely however, the concomitant presence of an oxidoreductase (OR), transaminase (TA), and hydrolase, may metabolize glyphosate in a three-step pathway, whereby glyphosate is first degraded into aminomethylphosphonic acid (AMPA) by OR, then converted into phosphonoformaldehyde through transamination by TA, and finally cleaved to produce phosphate and formaldehyde by a hydrolase (phosphonatase). Such degradation pathways were suggested for a range of glyphosate-metabolizing organisms [93, 95], and the results of our mass spectrometry work in combination with intracellular formaldehyde measurements, indicate that degradation by this pathway is predominant in our tested *Methylobacterium*. However, a search of the annotated genomes of *Methylobacterium* currently available through the National Center for Biotechnology Information (NCBI) did not indicate the presence of the glyphosate oxidoreductase (*goxA*) in the *Methylobacterium* strains used in sensitivity experiments. Evidence of mutation, whether random or intentional, enhancing or otherwise altering the sensitivity of Methylobacteriuum to glyphosate or GBH’s have not yet been reported.

Formaldehyde is a well-established metabolic by-product of normal enzymatic activity, with known toxicity to several critical components of the cell including proteins and nucleic acids [96–99]. In a range of bacteria, detoxification of formaldehyde is often achieved through a thiol-dependant pathway involving glutathione to produce the less cytotoxic, formate [100]. Methylotrophs, especially *Methylobacterium*, are unique in their ability to withstand transient intracellular loads of formaldehyde up to 1 mM which are produced as a result of single-carbon catabolism (methanol, methane, trimethylamine) [101]. In the cytoplasm, formaldehyde undergoes condensation with dephospho-tetrahydromethanopterin (dH4MPT) catalyzed by formaldehyde-activating enzyme (Fae) [101, 102]. As with other microorganisms, formaldehyde is eventually oxidized to produce formate. Crucially however, the dH4MPT complex appears to be responsible for handling both formaldehyde produced during growth, and formaldehyde contributions from degradation of xenobiotics [102–106].

In a study conducted by Bazurto et al., a previously unidentified member of the DUF336 domain family called *efgA* (enhanced formaldehyde growth), present in *Methylorubrum extorquens* (previously, *Methylobacterium extorquens*) and exclusive to methylotrophic taxa, was determined to encode EfgA, a formaldehyde sensor protein [107]. From the available evidence, accumulation and subsequent binding of formaldehyde to EfgA directly, results in reduced growth rate by triggering a reduction in global protein translation through the up-regulation of chaperone-encoding genes [107, 108]. This suggests that activation of EfgA by endogenous sources of formaldehyde works as part of a negative feedback loop to throttle enzymatic activity preventing overaccumulation of formaldehyde.

However, when glyphosate can gain entry to the cytosol through the lipid bilayer – mediated by the presence of a surfactant – the unchecked degradation of the herbicide in pathways governed by GOR-like enzymes, may lead to increases in formaldehyde levels as seen in *M. organophilum* (64.5 nM/mg) and *M. gnaphali* (72.7 nM/mg). As a result, the EfgA safety switch would respond to the increase in formaldehyde concentration and call for a reduction in translation. However, the enzymes involved in glyphosate degradation may not fall under the control of the EfgA feedback mechanism and proceed with glyphosate metabolism, unrestrained. In essence, the metabolism of glyphosate may contribute formaldehyde by several potential candidate pathways, including the oxidoreductase pathway indicated by our detection of the primary metabolite, AMPA. If these detoxification pathways do not sense the EfgA arrest signal, this molecular safety switch may become inadvertently and permanently flipped. Macromolecule damage may similarly occur if rapid detoxicification of formaldehyde to formate is attempted, resulting in a sharp change in intracellular pH. With the EfgA alarm activated, prolonged inhibition of growth and translation of critical proteins required for survival eventually reaches a point to which recovery from steadily accumulating formaldehyde and formate is irreversible and cell death becomes inevitable.

Although the presence of EfgA in all the strains used in experiments has not been confirmed through polymerase chain reaction (PCR), this unique signalling relay could explain the inconsistency surrounding cytotoxicity of glyphosate and GBH’s across many taxa of plant-associated microorganisms. The recurring debate regarding cytotoxicity of glyphosate formulations to microorganisms surrounds whether toxicity is due to the herbicide itself or to their co-formulants, notably the surfactants. In our work, neither Tween-20 in concentrations up to 4% or glyphosate in concentrations up to 0.1% showed cytotoxicity when administered alone. Yet, even at low concentrations of glyphosate (0.05%), the coadministration of an otherwise non-toxic surfactant, resulted in markedly reduced biomass. Moreover, if present, the prolonged activation of the EfgA molecular switch by formaldehyde contributed from glyphosate degradation, may also explain the observed negative impact on culturability when transferred to fresh medium after GBH exposure. This may be because, despite the fact that several of the *Methylobacterium* do likely contain a GS-EPSP synthase isoform, allosteric inhibition by glyphosate has been established as transitory and AAA biosynthesis should readily be restored after transfer to glyphosate-free growth media [109–111]. Additionally, several other microorganisms have demonstrated the ability to up-regulate the synthesis of EPSPS up to 30-fold, in an effort to compensate for blockade of the shikimate pathway caused by exogenous applications of glyphosate [112]. Taken together, the temporary and reversable disruption to AAA biosynthesis should be overcome with relative ease, however, the weak growth of *Methylobacterium* cells previously exposed to commercial GBH formulations and glyphosate-Tween20 mixes may be a result of more severe damage incurred to macromolecules by formaldehyde and a coinciding sustained arrest signal generated by EfgA. Importantly, in the absence of a timecourse study with greater granulatiry, the differences in detectable levels of intracellular formaldehyde and resultant culturability between strains may indicate variation in formaldehyde decomposition just as much as formaldehyde formation through degradation of intracellular glyphosate. While the presence of glyphosate-degradation pathways in other soil-borne or plant-associated microorganisms may result in transient toxicity, the reported near complete exclusivity of efgA and efgB loci to methylotrophs, may explain the apparent high sensitivity of *Methylobacterium* to GBH’s and glyphosate-Tween20 mixes.

### Implications

Host-plant pathogen protection following *Methylobacterium* inoculation was reported previously in studies involving potatoes and tomatoes, where the presence of *Methylobacterium* was also found to induce changes in plant microbiome composition [57, 59]. Next to resource competition caused by invasive weeds, infection and disease present a significant threat to crop development, for which future studies may find *Methylobacterium* to be particularly helpful.

Primarily, five species of pathogenic fungi are particularly problematic to field crop cultivation (*Phytophthora sojae, Phomopsis longicolla, Rhizoctonia solani, Pythium spp.*, and *Fusarium solani*) causing a range of diseases including seedling rot, seedling blight, and root rot. Interestingly, correlations between glyphosate application and the susceptibility of field crops including soybean to phytopathogens have previously been reported. For example, application of GBH’s has been linked to increased incidence of infection by *Phytophthora* spp [113, 114], *Glomus* spp. (Morandi 1989), and *Fusarium* spp [115]. Similarly, infection by *Gaeumannomyces tritici*, a known opportunistic pathogen affecting wheat, was found to increase following field pre-treatments with glyphosate [116, 117]. Use of glyphosate to control weed cover in barley was found to result in significant colonization of germinating seed by *Pythium* spp [118], and *Fusarium culmorum* [119, 120], causing poor crop performance. Vigor of winter rape seeded in soil pre-treated with glyphosate for the control of invasive weeds including quack grass, was clearly diminished with yields reduced by three-fold relative to untreated conditions [121]. Infection by *Fusarium* was also documented to increase in common waterhemp (*Amaranthus rudis*) following glyphosate treatment where survivability recorded for both glyphosate-sensitive (GS) and glyphosate-resistant (GR) varieties were reduced when grown in non-sterile (NS) soils relative to sterile conditions (GS: 29% reduced to 10% and GR: 83% reduced to 61%) [122]. Nearly identical infection dynamics were also documented in TopCrop beans and McIntosh Apples (*Malus domestica* Borkh.) where seedlings were treated with glyphosate and subsequently grown in both sterile and non-sterile conditions. The LD_50_ for glyphosate was markedly decreased in the presence of infections caused by *Pythium* spp. and *Fusarium* spp. in McIntosh seedlings (unchallenged: 40 µg, pathogen challenge: 10–15 µg) [123]. Furthermore, increases in disease incidence was associated with glyphosate application in more unique and geographically diverse crop systems including: banana [124], canola [125], cotton [126], maize [127], sugar beet [128], and tomato [129]. Studies examining the soybean rhizosphere in response to application of GBH’s also indicate significant disruption to population and diversity [130], including certain members responsible for reducing manganese (Mn) and secreting indole acetic acid (IAA) [131]. Importantly, both studies report higher incidence of disease caused by *Fusarium* spp. in soybean following glyphosate application, relative to controls [130, 131].

Several hypotheses surrounding the increased incidence of fungal disease in plants following glyphosate application have been proposed and include; (a) pathogen exposure to glyphosate predisposes the infectious agent to exogenous synthetic chemicals heightening virulence and fungicide resistance, (b) glyphosate may provide an alternate carbon source for pathogens, and (c) glyphosate-induced death of problematic weeds that act as reservoirs for certain phytopathogens cause sudden increases to field colony density during decay [132]. In addition to these, we propose that glyphosate may harm components of the phyllosphere, including the *Methylobacterium*, which prevent infection irrespective of the GR status of the host. Combined with the results presented herein, the relevant literature supports our theory that GBH’s may increase crop susceptibility to infections by selectively attenuating the protective effects of *Methylobacterium* colonization. Our work also demonstrates that even the presence of the relatively non-toxic surfactant, Tween20, can disrupt the growth of *Methylobacterium*. This is supported by the fact that, in addition to soybean, the *Methylobacterium* genus comprises a major part of the phyllosphere in several of the same food crop systems that exhibit increased susceptibility to infectious disease when exposed to GBH’s including: apple [133–135], wheat [62, 136, 137], tomato [138–140], hemp [141], cotton [142, 143], banana [144–147], and soybean [130, 131]. Importantly, the application of glyphosate has also been previously found to decrease host phytoalexin levels, even when attempting to elicit an immune response through deliberate pathogen challenge [148, 149]. As it so happens, *Methylobacterium* spp. have been documented to contribute to ISR and directly stimulate the synthesis of phytoalexins in plants [60, 149, 150].

## Conclusion

Our work on GBHs and surfactants indicate that glyphosate is toxic to *Methylobacterium*, and exibits bactericidal activity. While not harmful in its pure form alone, the toxic effects of glyphosate are observed when bacterial cell wall permeability is enhanced by the presence of a surfactant. The significance of our work is that while *Methylobacterium* species can be beneficial for plant growth, development, and disease protection, there are coinciding reports of greater disease activity when glyphosate is used for weed control. Our findings of heightened levels of intracellular formaldehyde in response to exogenously-applied GBH’s, with possible EfgA participation also lays the foundation for further investigation of the precise mechanics by which selective inhibition of discrete components of the phyllosphere is mediated. Our detection of AMPA, indicates conclusively that the tested *Methylobacterium* strains are metabolically active against glyphosate through an oxidoreductase-like pathway.

Continued investigation of PGPB such as *Methylobacterium* spp. presents a realistic path forward in the development of broad-spectrum biological fertilizers that not only make use of the natural biochemistry of the plant, but do so in an ecologically and toxicologically appropriate way. Future studies should involve targeted sequencing of glyphosate oxidoreductase (*goxA*) and enhanced formaldehyde growth (*efgA*) genes in *Methylobacterium* – and later, their knockouts – to compliment the metabolite results presented herein and further understand the mechanics of glyphosate toxicity in bacteria.

## Data Availability

The author confirms that all data generated or analysed during this study are included in this published article. Should any raw data files be needed in another format (raw, or unformatted) they are available from the corresponding author upon reasonable request.
